# Experiences of discrimination and their impact on healthcare utilization: non-uptake of covid-19 vaccination

**DOI:** 10.3389/fpubh.2025.1732845

**Published:** 2026-02-04

**Authors:** Nathalie Bajos, Alexis Spire, Antoine Sireyjol

**Affiliations:** 1 Institut de Recherche Interdisciplinaire sur les Enjeux Sociaux (IRIS), Institut National de la Santé et de la Recherche Médicale (INSERM), Aubervilliers, Paris, France; 2 CNRS, Paris, France

**Keywords:** COVID-19, discrimination, France, social inequalities, vaccination

## Abstract

**Objective:**

This article examines the relationship between experiences of discrimination and COVID-19 vaccine non-uptake, with particular attention to the domains in which discrimination occurs (healthcare, employment, housing, and public services) and to the frequency of such experiences.

**Methods:**

The analysis draws on the most recent wave of the Epidemiology and Living Conditions (EpiCov) cohort survey, conducted in October 2022, which included 65,403 adults living in metropolitan France.

**Results:**

Although the vast majority of the population in France ultimately received the COVID-19 vaccine, a significant minority remained reluctant to take advantage of this free and widely accessible intervention. Our findings indicate that past experiences of discrimination exerted both specific and cumulative effects on vaccination behavior: discrimination encountered in interactions with healthcare professionals and public services had a stronger influence on non-vaccination than discrimination related to employment or housing. Moreover, the frequency of discriminatory experiences was positively associated with vaccine non-uptake. Importantly, these associations were not limited to racialized minorities.

**Conclusions:**

By adopting a broad perspective on discrimination, the study demonstrates that feelings of social exclusion contribute to vaccine non-uptake across all social groups.

## Introduction

An extensive body of research has shown that vulnerable populations are consistently at greater risk of contracting SARS-CoV-2 and experiencing more severe symptoms (1). Ensuring equal access to COVID-19 vaccination remains a key public health challenge.

Numerous studies from different parts of the world have demonstrated that non-use of COVID-19 vaccination is more prevalent among populations with lower educational attainment, lower incomes, and among racialized minorities (2–10, 77).

The reasons behind non-use of vaccination have been the subject of extensive analysis. One set of factors concerns access to healthcare. Physical access to vaccination sites has been identified as a barrier to preventive practices (9, 11, 12). Financial constraints have also played a role, particularly in countries where vaccines were not provided free of charge (13). Other studies have emphasized that distrust toward healthcare professionals can also limit vaccine uptake (14).

Beyond these classic socio-economic determinants of healthcare non-use (15, 16), other distributed—namely, the unprecedented speed of its rollout, occurring barely a year after the pandemic began. In a context where governments and scientists assumed leading roles in managing the health crisis, it is crucial to analyze vaccination behavior in relation to levels of trust in governmental institutions (17, 18). In France, as in many other countries, the government strongly relied on scientific expertise to justify its pandemic response. Therefore, studying the impact of trust in government on vaccination behavior also entails accounting for trust in scientists (19).

Since disadvantaged social groups are often more distrustful of government institutions (20), the strong governmental involvement in vaccination programs—and the resulting politicization of these efforts—may have exacerbated social inequalities in vaccine uptake. Lack of trust in both government and scientific authorities has thus emerged as a significant barrier to COVID-19 vaccination (13, 21–24).

Another defining feature of the COVID-19 crisis has been the widespread circulation of opinions on social media, which fueled public controversies and the proliferation of conspiracy beliefs (25, 26). Several studies have shown that such conspiracy beliefs are associated with lower levels of vaccine uptake (27, 28).

Taking into account these various social determinants of non-uptake of vaccination calls for the examination of a dimension that remains relatively underexplored in the COVID-19 literature—namely, the impact of experiences of discrimination on vaccination behavior. Numerous studies have shown that populations subjected to racial discrimination are more likely to report general vaccine hesitancy (29–34). However, research examining the relationship between broader experiences of discrimination and vaccination behavior remains much more limited. In France, as in many other countries, those most reluctant to receive the vaccine were, as previously underlined, often drawn from the most socioeconomically disadvantaged populations, those with lower levels of educational attainment, and racialized minority groups (19, 35). These same social groups are also those most frequently exposed to various forms of discrimination (36, 57). However, what about individuals who have faced forms of discrimination other than those associated with race or socioeconomic status?

The World Health Organization defines structural discrimination as “the rules, norms, routines, patterns of attitudes and behaviors in institutions and other societal structures that represent obstacles to groups or individuals in achieving the same rights and opportunities as those available to most of the population,” and identifies it as a major determinant of health equity (16).

A growing body of research has established strong links between discrimination and health outcomes (37–40). Discrimination results from both individual behaviors and institutional practices, which in either case create barriers to healthcare access and are associated with lower quality of care. Structural discrimination also has broad and cumulative negative effects on key social determinants of health, including increased poverty and reduced access to education, employment, healthcare services, and safe and adequate living environments (41).

The few studies that have examined the impact of discrimination on vaccination practices have mostly limited their analyses to racialized minority groups. These studies attribute greater vaccine hesitancy among these populations to factors such as language barriers, fear of deportation, and limited access to vaccination (12), to experiences of everyday racism (42), or to heightened distrust of the healthcare system, partly resulting from encounters with healthcare professionals perceived as discriminatory (43, 44). Only one study—conducted by Wulkotte in Germany—has shown that experiences of discrimination are associated with lower vaccination uptake not only among migrant populations but also among non-migrants (45).

Experiences of discrimination that may influence vaccine non-use are not limited to belonging to racialized groups and can also be rooted in place of residence, particularly for individuals living in medical deserts (areas with severely limited access to healthcare services) (46). This spatial dimension of discrimination may intersect with racial criteria, as illustrated by public policies that have allowed environmental degradation in Overseas Territories, often at the expense of residents' health. One striking example is the widespread use of chlordecone, a carcinogenic pesticide banned in the United States as early as 1973 and in mainland France by 1990, but which continued to be used in the French Caribbean until 1993 to boost banana crop yields. The denunciation of the health and environmental consequences of chlordecone exposure has since become a symbol of protest against the discriminatory treatment of overseas populations in public policy decisions made from metropolitan France (47).

Discriminatory practices stemming from healthcare professionals' own judgments can also play a significant role in the quality of medical care provided. For instance, in clinical situations where symptoms and severity are identical—such as chest pain—men tend to be taken more seriously than women, and white patients more than those belonging to racialized minorities (48). Similarly, emergency physicians encountering Roma patients—who combine “ethnoracial otherness” with socioeconomic, residential, and administrative precarity—often attribute their more frequent use of hospital services to “cultural difference,” despite their reasons for visiting emergency departments being comparable to those of the general population (49). These discriminatory processes are frequently based on healthcare providers' perception of otherness, leading to mechanisms of delegitimization and differentiation (50). Such dynamics have also been observed in the management of hypertensive disorders during pregnancy among women of sub-Saharan African origin (51). Discrimination related to sexual orientation or gender identity can also have a significant impact on healthcare access (52, 53).

To better understand the relationship between discrimination and COVID-19 vaccination, we propose two central hypotheses. Structural discrimination occurs systematically across society and is embedded in dominant cultural norms, legal systems, and economic structures—not solely within the healthcare sector. Our first hypothesis is that the relationship between experiences of discrimination and non-vaccination may vary depending on the type of discrimination experienced (54). Such experiences may occur in interactions with healthcare professionals, but also in areas such as access to employment, housing, or public services, core domains where discriminatory practices are well documented (57). The key issue here is to examine how these experiences influence vaccination practices, with the understanding that discriminatory treatment does not have the same consequences for attitudes and practices depending on whether or not it occurs in relation to institutional representatives. Discrimination experienced within institutional settings may have a particularly strong impact on trust and compliance with public health recommendations.

Our second hypothesis posits that frequent exposure to discrimination reduces the likelihood of vaccine uptake. Experiencing discrimination undermines trust and confidence in a society's social, economic, political, and cultural systems (16). Discrimination acts as a social reminder that signals to affected individuals that they do not occupy the same place in society as others. As Pollak (55) showed in his study on gay men during the AIDS epidemic, the repeated reminder of belonging to a stigmatized minority can foster a sense of social exclusion that is incompatible with engagement in preventive health behaviors. We thus hypothesize the existence of a cumulative effect of discrimination on vaccine non-use.

Based on a large-scale quantitative survey conducted among the general population, this article aims to explore the relationship between experiences of discrimination and non-use of COVID-19 vaccination. Unlike previous studies that focus on specific subpopulations, our analysis considers a wide range of discriminatory contexts—including interactions with healthcare professionals, employment, housing, and public services—as well as the frequency of such experiences.

## Methodology

### Research protocol

The *Epidemiology and Living Conditions* (EpiCov) survey was developed in the context of the COVID-19 pandemic by the French National Institute of Health and Medical Research (Inserm) and the Directorate for Research, Studies, Evaluation, and Statistics (DREES), in collaboration with Santé publique France (SpF) and the National Institute of Statistics and Economic Studies (Insee).

The survey methodology has been described in detail elsewhere (56), and we will only summarize here the main methodological features relevant to this article. Survey participants were randomly selected from the Fidéli national administrative database (Fichier démographique sur les logements et les individus), which is based on tax records.

The survey was conducted in four waves. The first three took place in May 2020, November 2020, and Summer 2021. The fourth wave—the focus of the present study—was conducted between September 12 and December 5, 2022.

Among the 85,032 individuals who participated in the first three waves, 65,403 responded to the fourth wave, which included a specific module on experiences of discrimination. For this analysis, the study population consists of the 63,377 individuals aged 18 and over as of January 1, 2022, residing in mainland France in private households, and having responded to all the survey questions used in the analysis—representing 97% of respondents to the fourth wave.

### Analytical variables

#### Sociodemographic characteristics

The analyses included the following sociodemographic variables: sex, age, educational attainment, standard of living (measured by income level), and urban area size.

#### Racialized minority status

Membership in a racialized minority group was defined based on the country of birth and nationality of the respondent (*ego*) and their parents. This classification draws on typologies developed in the *Trajectories and Origins* surveys conducted in 2008 and 2019 (57), as well as on the framework proposed in the analysis of discrimination in France using data from the *Défenseur des droits* (58).

We distinguished the following categories:

- Majority population: individuals who are neither immigrants nor descendants of immigrants, and who were neither born in French overseas departments and regions (DROM) nor descended from individuals born in DROM.
- DROM population: individuals born in DROM or descendants of individuals born in DROM.
- Non-racialized immigrants: immigrants from countries in Europe, the Americas, Australia, or New Zealand.
- Racialized immigrants: immigrants from North Africa, Sub-Saharan Africa, Asia, or the Middle East.
- Descendants of non-racialized immigrants: individuals whose parents immigrated from Europe, the Americas, Australia, or New Zealand.
- Descendants of racialized immigrants: individuals whose parents immigrated from North Africa, Sub-Saharan Africa, Asia, or the Middle East.

#### Vaccination status

There are multiple definitions of what constitutes a complete COVID-19 vaccination schedule, and these may vary across health authorities and expert bodies. In this study, which focuses on non-use of vaccination, we defined as unvaccinated all individuals who reported not having received any dose of a COVID-19 vaccine, regardless of whether they had previously tested positive for the virus.

#### Experiences of discrimination

The discrimination module was introduced with a statement to participants: “*Let's move on to another topic, unrelated to the coronavirus,”* in order to exclude from the scope of responses any perceived discrimination related to the health pass or vaccination pass.

The questionnaire captured the frequency, perceived reasons, and contexts of discrimination. Respondents were first asked about the frequency of unequal treatment or discrimination over the past 5 years:

“*In the past five years, do you think you have experienced unequal treatment or discrimination? This could have occurred in relation to employment, housing, with a healthcare provider, in hospital, at school, in public services, in the street, etc.”*

The response options were:

1. Often
2. Sometimes
3. Never

Next, respondents were asked about the perceived reasons for such discrimination:

“*In your opinion, was it mostly due to …”*

- Your age
- Your sex (being a man or a woman)
- Your health status or a disability
- Your skin color
- Your origins or nationality
- The place where you live, or the reputation of your neighborhood
- Your sexual orientation
- Your religion
- Your weight
- Another reason
- You don't know
- You prefer not to say

Finally, respondents were asked where these discriminatory experiences occurred:

“*Where did this experience (or these experiences) take place?”*

- In a professional context (e.g., job search, at the workplace)
- When looking for housing
- With a healthcare professional or in a hospital
- In interactions with a public service or administration (e.g., town hall, post office, employment office, etc.)
- In another context

### Analytical strategy

We first present univariate analyses of both non-use of vaccination (Table 1) and experiences and perceived reasons for discrimination (Tables 2 and 3). Non-responses (which never exceed 2%) were excluded from all analyses. “Don't know” responses are reported in the descriptive statistics but not included in the multivariate models.

**Table 1:** Non-uptake of COVID-19 vaccination by social characteristics (row percentages).

**Variables**	**No dose**	**Total numbers**
Total	7.1%	63,377
**Gender** ^*^		
Men	7.7	27,524
Women	6.5	35,853
**Age** ^*^		
18–24	8.5	5,004
25–34	10.8	6,166
35–44	10.1	9,700
45–54	8.4	12,536
55–64	5.6	13,230
65–74	3.3	11,438
75+	3.1	5,303
**Degree** ^*^		
No diploma	7.2	2,518
Lower secondary	5.8	5,528
Technical diploma	8.4	11,354
High school diploma	8.7	12,809
Post-secondary	7	19,534
Postgraduate degree	3.8	11,634
**Standard of living** ^*^		
D1	13.7	3,848
D2–D3	10.5	6,563
D4–D5	7.7	8,986
D6–D7	6.1	13,103
D8–D9	3.8	18,576
D10	2.4	10,813
**Area size** ^*^		
Rural	6.8	15,029
< 20,000 inh	7.4	10,956
20,000–100,000 inh	7.8	8,214
>100,000 inh	6.9	27,027
**Racialized minority status** ^*^		
Majority population	6.1	53,113
DROM population	15.6	675
Non-racialized immigrants	7.2	3,404
Racialized immigrants	16.1	1,768
Descendants of non-racialized immigrants	7.2	1,863
Descendants of racialized immigrants	9.8	1,687

Source: Enquête EpiCoV Inserm-Drees, vague 4, Sept–Dec 2022.

Weighted percentages, raw counts.

^*^Denotes statistical significance at the 5% level.

**Table 2:** Experiences of discrimination in the past 5 years by social characteristics (% by row).

**Variables**	At least one^**^discrimination	**Health**	**Employment**	**Housing**	**Publics Services**
Total	16.3	2.6	7.3	1.7	3.3
**Gender** ^*^					
Men	14.2	1.9	5.9	1.5	3.2
Women	18.3	3.3	8.7	1.8	3.3
**Age** ^*^					
18–24	20.6	2.5	8.4	1.6	3
25–34	25.3	4.5	13.4	3.9	5.3
35–44	23.7	3.9	12.4	3.1	5.5
45–54	19.6	2.7	9.3	1.9	3.5
55–64	14.1	1.8	6.6	0.8	2.7
65–74	6.7	1.3	1.0	0.3	1.5
75+	4.7	1.7	0.1	0.0	1.1
**Degree** ^*^					
No diploma	13.2	2.6	3.8	1.6	3.1
Lower secondary	11.6	1.9	3.2	0.7	1.9
Technical diploma	13.7	2.1	5.3	1.3	2.8
High school diploma	19	2.9	8.4	2.1	3.9
Post-secondary	19.4	3.2	10.5	2.0	4.0
Postgraduate degree	18.9	2.7	11.3	2.0	3.2
**Standard of living** ^*^					
D1	23.9	4.2	9.6	4.2	5.6
D2–D3	20.6	3.5	8.5	2.8	4.4
D4–D5	16.8	2.5	7.3	1.6	3.3
D6–D7	14.4	2.2	7.0	0.9	2.7
D8–D9	12.4	1.9	6.2	0.7	2.0
D10	10.5	1.3	5.1	0.4	1.6
**Area size** ^*^					
Rural	13.0	2.3	5.7	0.7	2.5
< 20,000 inh	14.4	2.3	6.1	1.1	3.0
20,000–100,000 inh	16.5	2.8	6.4	1.3	3.3
>100,000 inh	18.5	2.7	8.9	2.4	3.6
**Racialized minority status** ^*^					
Majority population	13.9	2.3	6.4	0.9	2.5
DROM population	32.1	4.2	16.6	6.1	5.7
Descendants of non-racialized immigrants	14.2	2.5	6.2	1.0	2.8
Descendants of racialized immigrants	35.8	5.4	17.1	7.0	9.7
Non-racialized immigrants	14.6	1.8	5.1	1.3	3.3
Racialized immigrants	31.3	4.5	12.1	6.6	7.4

Source: EpiCoV Survey, Inserm-DREES, Wave 4, Sept–Dec 2022.

Interpretation: 16.3% of respondents reported having experienced at least one form of discrimination, and 2.6% reported at least one experience of discrimination in the healthcare domain. Percentages are weighted; counts are unweighted.

^*^Denotes statistical significance at the 5% level.

^**^The sum of reported experiences does not equal 16.3% due to instances of multiple forms of discrimination, experiences that span several domains, or the occurrence of discrimination in domains beyond the four listed.

**Table 3:** Reported grounds for discrimination by type of discriminatory experience (% by column)^*^.

**Reasons**	**Health professionals**	**Other discrimination**	**Total (numbers)**
Age	19.2	16.6	17 (1,763)
Gender	28.3	25.2	25.7 (3,196)
Health status	20.9	9.2	11 (913)
Skin color	11.4	12.2	12.1 (782)
Origine	18.5	20.1	19.8 (1,305)
Area size	7.5	4.9	5.3 (387)
Sexual orientation	3.4	2.7	2.8 (255)
Religion	10.6	7.3	7.9 (462)
Weight	20.6	8.5	10.4 (997)
Other	25.0	23.9	24 (2,431)
Do not know	29.2	33.5	32.8 (3,194)

Source: EpiCoV, wave 4, Sept–Dec 2022.

Interpretation: 19.2% of individuals reporting discrimination in their interactions with healthcare professionals indicated that the incident was related to their age. Percentages are weighted; raw counts are shown.

The sum of reported reasons for discrimination within a given domain exceeds 100%, as respondents could select multiple reasons.

^*^Denotes statistical significance at the 5% level.

In a second step, we conducted logistic regression models to examine the association between experiences of discrimination and non-use of COVID-19 vaccination, adjusting for social characteristics.

- Sociodemographic variables (Table 4, first column).
- Sociodemographic variables + type of discrimination (Table 4, second column).
- Sociodemographic variables + frequency of discrimination (Appendix 1).
- Sociodemographic variables + type and frequency of discrimination (Appendix 2).

**Table 4:** Factors associated with non-vaccination according to the inclusion of type of discrimination (logistic regression models).

	**%**	**Sociodemographic characteristics**		**Sociodemographic characteristics** + **type of discrimination**	
**Age**	**%**	**OR**	**95%CI**	**OR**	**95%CI**
18–24	8.5	1.42^*^	[1.18–1.70]	1.38^*^	[1.15–1.66]
25–34	10.8	2.78^*^	[2.36–3.27]	2.59^*^	[2.20–3.06]
35–44	10.1	2.44^*^	[2.10–2.83]	2.30^*^	[1.97–2.67]
45–54	8.4	1.67^*^	[1.44–1.94]	1.62^*^	[1.40–1.88]
55–64	5.6	1.34^*^	[1.15–1.56]	1.32^*^	[1.13–1.53]
65–74	3.3	ref		ref	
75+	3.1	0.70^*^	[0.55–0.88]	0.69^*^	[0.55–0.88]
**Gender**					
Men	7.7	1.10^*^	[1.01–1.19]	1.14^*^	[1.05–1.24]
Women	6.5	Ref		Ref	
**Standard of living**					
D1	13.7	3.74^*^	[3.09–4.52]	3.51^*^	[2.90–4.25]
D2–D3	10.5	3.16^*^	[2.65–3.78]	3.02^*^	[2.53–3.62]
D4–D5	7.7	2.33^*^	[1.95–2.78]	2.28^*^	[1.91–2.72]
D6–D7	6.1	1.83^*^	[1.54–2.18]	1.80^*^	[1.51–2.13]
D8–D9	3.8	1.36^*^	[1.15–1.61]	1.35^*^	[1.14–1.59]
D10	2.4	Ref		Ref	
**Degree**					
No diploma	7.2	0.99	[0.82–1.20]	1.01	[0.83–1.23]
Lower secondary	5.8	0.86	[0.73–1.02]	0.87	[0.74–1.03]
Technical diploma	8.4	0.98	[0.87–1.11]	0.99	[0.88–1.12]
High school diploma	8.7	Ref		Ref	
Post secondary	7	0.79^*^	[0.71–0.88]	0.79^*^	[0.71–0.88]
Postgraduate degree	3.8	0.52^*^	[0.45–0.60]	0.51^*^	[0.44–0.60]
**Racialized minority status**					
Majority population	6.1	Ref		Ref	
DROM population	15.6	2.33^*^	[1.79–3.02]	2.30^*^	[1.77–2.99]
Non-racialized immigrants	7.2	1.17	[0.99–1.40]	1.17	[0.99–1.39]
Racialized immigrants	16.1	2.06^*^	[1.74–2.44]	1.93^*^	[1.63–2.30]
Descendants of non-racialized immigrants	7.2	1.21	[0.97–1.53]	1.21	[0.96–1.52]
Descendants of racialized immigrants	9.8	1.31^*^	[1.06 - 1.61]	1.26^*^	[1.02–1.55]
**Area size**					
Rural	6.8	1.27^*^	[1.14–1.40]	1.28^*^	[1.16–1.42]
< 20,000 inh	7.6	1.19^*^	[1.06–1.33]	1.21^*^	[1.08–1.35]
20,000–100,000 inh		1.13^*^	[1.00–1.28]	1.14	[1.00–1.29]
>100,000 inh	6.7	Ref		Ref	
**Type of discrimination**					
**Employment**					
No	6.8	–		Ref	
Yes	10.7	–		1.20^*^	[1.06–1.37]
**Housing**					
No	6.9	–		Ref	
Yes	16.5	–		1.02	[0.76–1.36]
**Health**					
No	6.8	–		Ref	
Yes	19.9	–		2.80^*^	[2.36–3.32]
**Public services**					
No	6.8	–		Ref	
Yes	16.4	–		1.36^*^	[1.13–1.64]

Source: EpiCoV Survey, Inserm-Drees, wave 4, Sept–Dec 2022. Weighted percentages, unweighted counts.

Interpretation: 13.7% of individuals in the lowest decile of standard of living (D1) were unvaccinated. In the first column, which does not account for discrimination, individuals in this group had 3.7–4 times higher odds of non-vaccination compared with those in the highest decile (D10). This odds ratio decreases to 3.51 in the second column (adjusted for types of discrimination).

^*^Denotes statistical significance at the 5% level.

To assess whether the association between discrimination and non-vaccination is present across all social groups, we conducted an analysis of factors associated with non-vaccination using a logistic regression model that included an interaction term between discrimination and racialized minority status (Appendix 3).

We conducted a sensitivity analysis by excluding respondents who reported having experienced discrimination but did not select any of the proposed reasons as a distinct group—possibly indicating they did not experience discrimination in the same way as others. The results (Appendix 4) show that all the odds ratios remained statistically significant.

Finally, although the discrimination module was explicitly introduced with the phrase “*Let's move on to another topic, unrelated to the coronavirus,”* we cannot rule out the possibility that some respondents who reported having experienced discrimination—but did not select any of the proposed reasons (e.g., age, sex, health status, place of residence, skin color, etc.)—may have been referring to their unvaccinated status. To address this, we compared the socio-demographic characteristics of those who selected at least one proposed reason for discrimination with those who selected none, and we also compared their attitudes toward vaccination using two indicators:

1. “The government is hiding information about the vaccine”
2. “We lack sufficient hindsight on the vaccine's effects”

The results (not shown) indicate that individuals who did not report any specific reason for their discrimination experiences were primarily men over age 50 living in rural areas, and they did not express any particular mistrust or hostility toward vaccination.

All percentages are weighted to account for the survey design and response rates, while raw numbers reflect the actual number of respondents. Univariate analyses use a 0.05 significance threshold from Chi-square tests, and multivariate analyses interpret significant differences based on 95% confidence intervals.

## Results

### Social mapping of COVID-19 vaccine non-use

In the fall of 2022, 7.1% of respondents in the EpiCov survey reported having received no dose of a COVID-19 vaccine (Table 1).

Men were slightly less likely to be vaccinated than women. Individuals who had not received any COVID-19 vaccine doses were predominantly aged 25–44 years, while older age groups were significantly underrepresented among the unvaccinated. Vaccine non-uptake was strongly and linearly associated with household financial resources: while 13.7% of individuals in the bottom 10% of the income distribution were unvaccinated, this proportion steadily decreased to 2.5% among those in the highest income decile.

Disparities were even more pronounced when considering racial origin. Among individuals born in, or descended from someone born in, French overseas territories (DROM), 15.6% had not been vaccinated. Similarly, 16.1% of descendants of racialized immigrants remained unvaccinated, compared to 9.8% of racialized immigrants themselves and 6.1% of individuals born in mainland France to French-born parents (the majority population).

### Socially situated experiences of discrimination

According to the EpiCoV survey, individuals reporting experiences of discrimination represented a minority of the general population, though some may have experienced discrimination in multiple contexts. In total, 16.3% of respondents answered affirmatively to the question:

“*In the past five years, do you think you have experienced unequal treatment or discrimination? This could have occurred in employment, housing, with a healthcare professional, in a hospital, at school, with public services, in the street, etc.”*

Among those reporting discriminations, 11.2% stated that such experiences occurred “sometimes” and 18.2% reported they occurred “often.”

Discrimination was most frequently reported in the employment sector (7.3% of the total population), followed by interactions with public services (3.3%), healthcare professionals (2.6%), and access to housing (1.7%) (Table 2).

### Characteristics of individuals reporting experiences of discrimination

The profile of individuals reporting experiences of discrimination reveals distinct social patterns, with a significant difference by gender: 14.0% of men and 18.2% of women aged 18 and older reported having been discriminated against. Discrimination was more frequently reported by younger and more educated individuals: 25% of respondents aged 25–34 reported experiencing at least one instance of discrimination in the past 5 years, a proportion that reached 19.3% among those with a post-secondary degree (equivalent to 2–4 years beyond the baccalaureate) (Table 2).

Despite this socially differentiated awareness or recognition of discriminatory treatment, it was individuals with the lowest incomes—and more so those belonging to racialized minority groups—who most frequently reported such experiences: 23.7% of individuals among the bottom 10% income group reported at least one experience of discrimination, compared with 10.5% among those in the top 10%. This figure rose to 36% among descendants of racialized immigrants. In contrast, non-racialized immigrants and their descendants reported experiences of discrimination at rates similar to the majority population (14%). People living in cities with more than 100,000 inhabitants were also particularly affected, with 20.6% reporting at least one experience of discrimination.

The social profile of those reporting discrimination varied only slightly across different domains. While women consistently reported discrimination more often than men, this difference was especially pronounced in healthcare (3.3% vs. 1.9%) and employment (8.7% vs. 5.9%), where gender-based discrimination was more frequently cited. In the employment domain, the most affected groups included intermediate professionals (8.8%), employees (8.2%), executives (7.8%), and individuals holding post-secondary degrees (10.5% for Bac+2 to Bac+4; 11.3% for Bac+5).

In interactions with public services, racialized minorities reported particularly high levels of discrimination: 9.7% of descendants of racialized immigrants and 7.4% of racialized immigrants reported such experiences, compared with 2.5% of the majority population. Similar patterns were observed in the housing sector, where 7.0% and 6.6% of these two groups, respectively, reported discrimination, vs. 0.9% among the majority population.

When looking at the reasons for discrimination reported by respondents—after the survey clarified that this section was unrelated to the COVID-19 pandemic—the most frequently cited reason was gender (Table 3). Origin or skin color origin was also often mentioned, by roughly one in five respondents. With the exception of health status, which was more frequently cited in cases involving healthcare professionals, reported motives varied little across domains. Finally, nearly one in three respondents said they did not know the reason they had been discriminated against (Table 3).

### Discrimination and vaccination

Participants reporting no experiences of discrimination had a 6.0% rate of not receiving any vaccine dose, compared to 10.7% among those discriminated against in employment, 16.4% among those discriminated against during interactions with public services, 16.5% among those discriminated against in housing access, and 19.9% among those experiencing discrimination in healthcare settings (Table 4).

The strong association between discrimination experienced in healthcare settings and non-vaccination prompts a closer look at the profile of individuals reporting this type of discrimination (Table 2). Discrimination in healthcare was reported more often by women (3.3%) than by men (1.9%), with younger individuals also showing a higher prevalence of such experiences. Individuals aged 25 to 44 appeared most exposed to healthcare-related discrimination (4.5% of those aged 25–34 and 3.9% of those aged 35–44, compared to 1.3% of those aged 65–74), despite this being a life stage with generally fewer medical consultations. Members of racialized minority groups were also more affected: 4.2% of people from overseas territories (DROM) reported medical discrimination, rising to 4.5% for racialized immigrants and 5.4% for their descendants.

Since the social characteristics of non-vaccinated individuals and those reporting discriminations partially overlap—with the notable exception of women, who reported discrimination more frequently yet had higher vaccination rates—it is important to assess the relationship between discrimination and vaccine uptake while controlling for other social variables.

Multivariate analysis results first show that individuals situated lower in the social hierarchy were less likely to be vaccinated (Table 4, first column). Specifically, the poorest decile of income earners had an odds ratio (OR) of 3.74 (95% CI: 3.09–4.52) for non-vaccination. Similarly, natives or descendants of DROM populations and second-generation racialized minorities had lower vaccination uptake. People living in rural areas and in towns with fewer than 20,000 inhabitants were also less likely to be vaccinated: OR = 1.27 (95% CI: 1.14–1.40) and 1.19 (95% CI: 1.06–1.33), respectively.

When discrimination across different social spheres was examined (Table 4, second column), lower vaccination rates were observed for all types of discrimination except housing-related discrimination. People discriminated against in employment had 1.2 times higher odds of non-vaccination (95% CI: 1.06–1.37). The odds ratio for non-vaccination rose to 1.36 (95% CI: 1.13–1.64) when discrimination occurred in interactions with public services. The association was strongest for discrimination experienced in healthcare or hospital settings (OR = 2.80; 95% CI: 2.36–3.32).

Next, a cumulative association was observed between discrimination experiences and vaccination behavior (Appendix 1). Participants with intermittent experiences of discrimination were less likely to be vaccinated against COVID-19 (OR = 1.78; 95% CI: 1.61–1.96), independently of social characteristics, and those frequently experiencing discrimination had an even higher likelihood of non-vaccination (OR = 2.41; 95% CI: 2.02–2.86).

Finally, a comparison of the logistic regression models excluding discrimination (Table 4, first column) with the model including both the frequency and context of discrimination (Appendix 2) showed that the social factors associated with non-vaccination remained largely unchanged. All odds ratios remained significant, with minimal variation in the confidence intervals. The similarity in odds ratios across models indicates that discrimination experiences do not fully explain vaccination disparities between social groups. The results confirm that discrimination in interactions with healthcare professionals has the strongest impact on non-vaccination. For this type of discrimination, experiencing it frequently rather than occasionally does not further change the likelihood of non-vaccination.

## Discussion

Although COVID-19 vaccine uptake was high among the population in France, a significant minority remained reluctant to take up this free and widely accessible intervention. Our results indicate that experiences of discrimination are associated with specific and cumulative differences in vaccination practices: discrimination encountered during interactions with healthcare professionals, and to a lesser extent with public services, was more strongly associated with non-vaccination than discrimination related to employment or housing. Importantly, these associations were observed across all groups, whether racialized or not, as shown by the analysis of factors associated with non-vaccination that accounts for the interaction between discrimination and racialized minority status, even though experiences of discrimination alone do not fully explain the lower vaccination rates among racialized groups.

The social profile of unvaccinated individuals in autumn 2022 was similar to that observed during the previous wave of the EpiCov survey conducted in summer 2021 (59). This stability suggests that vaccine non-uptake does not primarily reflect delays in access or temporary barriers, but rather represents a sustained pattern of non-vaccination from the vaccine's widespread availability in June 2021 through the administration of the questionnaire in autumn 2022. The results indicate that this non-use is partly explained by lived experiences of discrimination occurring across various social spheres (employment, health, public services) and linked to diverse grounds (sex, age, weight, etc.).

Whereas the existing studies on the relationship between discrimination and vaccine non-uptake have limited their analyses to racialized individuals disadvantaged by the healthcare system (12, 42, 60–63), our approach considers multiple types of discrimination across different social domains. Our findings reveal that discrimination experienced within healthcare settings, during interactions with public services, and in the workplace, each increases the likelihood of being unvaccinated, indicating that discrimination experiences may play a role in shaping vaccine acceptance. More specifically, discrimination in housing did not appear to affect vaccination practices, whereas the association was notably strong for discrimination in healthcare and, to a lesser extent, in public service contexts. In other words, discrimination originating from social or state institutions exerted the greatest impact on vaccine non-uptake.

One might hypothesize that these results primarily reflect a heightened distrust of public authorities among the least vaccinated social groups, both in general and specifically regarding their management of the COVID-19 pandemic. However, analyses controlling for trust in government showed that the associations with experiences of discrimination remained consistent, with significant odds ratios (see Appendix 5). The strong influence of discrimination experiences in healthcare—and to a lesser extent in public services—may also relate to the unique status of the COVID-19 vaccination campaign, which was both recommended by healthcare professionals and heavily promoted by public authorities.

This finding aligns with previous research showing that trust in healthcare professionals plays a key role in vaccine hesitancy (8, 14, 64). Individuals reporting discrimination tend to have lower trust in these actors (65). However, the concept of trust in the healthcare system is complex and difficult to interpret (66), as perceptions vary widely depending on geographical location (with significant territorial inequalities), institutional status (public hospital vs. private clinic), and professional status (private practitioner vs. hospital-based clinician).

The results also highlighted the cumulative influence of discrimination on vaccine non-uptake, which increased with the frequency of discriminatory experiences—except in cases involving health professionals, where even a single encounter appears sufficient to generate distrust toward the healthcare system. In the literature, this association has primarily been demonstrated for individuals belonging to racialized minorities: racial discrimination is presented as a key factor explaining the lack of trust in health authorities among these minorities, and this mistrust is itself linked to lower vaccination uptake (30, 42, 67). Our findings confirm this pattern: racialized minorities were particularly affected by discrimination from institutional actors and were also among the groups with the lowest vaccination rates. While immigrants from racialized minority groups exhibited lower vaccination rates compared to the majority population, vaccine non-uptake was even more pronounced among their descendants.

Interestingly, although women reported higher rates of discrimination than men, their COVID-19 vaccine uptake was higher. A more detailed analysis reveals that this exception is not truly one. Among women reporting no discrimination in the 5 years preceding the survey, 5.7% remained unvaccinated, whereas this proportion rose to 9% for those discriminated against in the workplace, 12.3% for those facing discrimination in contact with public services, and 13.9% in healthcare settings. Thus, even among women, a gradient was observed according to the sphere in which discrimination occurred, in relation to vaccination status. Even though women face high discrimination rates, their strong pro-vaccine behavior tied to their gender identity (likely linked to social norms of care-giving or higher engagement with preventive health) outweighs the negative impact of their discrimination experiences, resulting in a net positive uptake rate.

Multivariate analysis showed that the association between experiences of discrimination and vaccine non-uptake was not limited to racialized minorities but was observed across all social groups, consistent with findings from Wulkotte's study (45). Hence, adopting a broad spectrum of discrimination experiences makes it possible to show that their consequences are not limited to racialized populations. However, since these minorities are more frequently exposed to discrimination overall, they are consequently more likely to be affected by the link between discrimination and vaccine non-uptake.

## Limitations

As with any general population survey, the EpiCov study has limitations that should be acknowledged. Homeless individuals were not included in the sampling frame, as they are not affiliated with the tax registry that served as the sampling base for this survey. Yet, these individuals face major barriers to accessing healthcare in general and vaccination in particular (11, 68). Consequently, the rate of vaccine non-uptake is likely underestimated, and the social determinants of vaccine non-uptake within these groups may differ from those identified in this study.

Moreover, to avoid recall bias regarding distant episodes of discrimination, we limited the analysis to events occurring within the past 5 years without further chronological specification. However, we cannot exclude that other significant events, such as the deterioration of a close relative's health due to SARS-CoV-2 infection, may have occurred since the discrimination episode and also influenced vaccination decisions.

It is also important to note that our analyses rely on self-reported experiences of discrimination. Some may argue that questionnaire responses do not necessarily reflect the objective reality of lived experiences. This methodological challenge is well-known among researchers in this field (69). We adopted formulations used in specific French surveys on discrimination, which have been shown to produce results consistent with records of discrimination by institutional bodies tasked with combating discrimination, such as the Defender of Rights (58).

Finally, we did not account for the sources of information individuals used regarding vaccination. Misinformation campaigns have been widespread in France and other countries, especially on social media (70). It can be assumed that socially disadvantaged individuals were most exposed to such misinformation (10). Since vaccine uptake is lowest in these groups, including this factor might have enhanced our understanding of the drivers of vaccine non-uptake.

## Conclusion

Considering a broad spectrum of discrimination types and their association with vaccine non-uptake has revealed the structuring role of experiences perceived as injustices, particularly in encounters with institutions meant to guarantee and embody equal treatment. Our findings confirm that inequalities in healthcare access—even when services are free and widely available, as was the case with COVID-19 vaccination—cannot be attributed solely to beliefs or cultural differences. Instead, they may be associated with discriminatory practices that have shaped the worldview of those affected.

This result nuances studies that interpret vaccine hesitancy solely as a consequence of misinformation (71) or susceptibility to conspiracy theories (72). The significant role of discrimination, particularly in healthcare settings, underscores the importance of past experiences in shaping relationships with the body and medicine. Health inequalities are not only a striking manifestation of the embodiment of social structures (73) but also the product of unequal and discriminatory experiences in care and treatment.

COVID-19 vaccination has prevented approximately 160,000 deaths and 1.5 million hospitalizations in France (74), and nearly twenty million deaths worldwide (75). These facts should be considered when designing future vaccination campaigns (76). Moreover, integrating lived experiences with state and healthcare institutions may help explain observed social disparities in the uptake of preventive measures such as cervical or colorectal cancer screening and other widely accessible health goods that remain unevenly utilized.

## Data Availability

The original contributions presented in the study are included in the article/supplementary material, further inquiries can be directed to the corresponding author.
