# Supplementary material for: Experiences of discrimination and their impact on healthcare utilization: non-uptake of covid-19 vaccination
Source: Front Public Health. 2026 Feb 4;13:1732845. doi: 10.3389/fpubh.2025.1732845 (PMC12915329; doi:10.3389/fpubh.2025.1732845)
Supplement: Supplementary file 1 [file Table_1.pdf]

Appendix 1: Factors associated with non-vaccination including the frequency of discrimination (logistic regression model)

Variables	%	OR [95% CI]
Age		
18 - 24	8.5	1.29 [1.07 - 1.55]
25 - 34	10.8	2.48 [2.10 - 2.93]
35 - 44	10.1	2.21 [1.90 - 2.57]
45 - 54	8.4	1.55 [1.33 - 1.80]
55 - 64	5.6	1.27 [1.10 - 1.48]
65 - 74	3.3	ref
75 +	3.1	0.70 [0.55 - 0.89]
Gender		
Men	7.7	1.15 [1.06 - 1.24]
Women	6.5	ref
Standard of living		
D1	13.7	3.49 [2.88 - 4.22]
D2-D3	10.5	2.99 [2.50 - 3.57]
D4-D5	7.7	2.24 [1.88 - 2.68]
D6-D7	6.1	1.79 [1.50 - 2.12]
D8-D9	3.8	1.34 [1.13 - 1.59]
D10	2.4	ref
Degree		
No diploma	7.2	1.04 [0.86 - 1.26]
Lower secondary	5.8	0.88 [0.74 - 1.04]
Technical diploma	8.4	1.00 [0.88 - 1.12]
High school diploma	8.7	ref
Post secondary	7	0.80 [0.71 - 0.89]

Postgraduate degree	3.8	0.52	[0.44 - 0.60]
Racialized minority status			
Majority population	6.1	ref	
DROM population	15.6	2.15	[1.66 - 2.79]
Non-racialized immigrants	7.2	1.17	[0.98 - 1.39]
Racialized immigrants	16.1	1.83	[1.54 - 2.17]
Descendants of non-racialized immigrants	7.2	1.20	[0.95 - 1.51]
Descendants of racialized immigrants	9.8	1.17	[0.95 - 1.44]
Area size			
Rural	6.8	1.29	[1.17 - 1.43]
< 20 000 inh	7.6	1.21	[1.08 - 1.35]
20 000 - 100 000 inh		1.14	[1.00 - 1.29]
> 100 000 inh	6.7	ref	
Frequence of discrimination			
Never	6.0	ref	
Sometimes	11.2	1.78	[1.61 - 1.96]
Often	18.2	2.41	[2.02 - 2.86]

Source: *EpiCoV Survey, Inserm-Drees, wave 4, Sept–Dec 2022. Weighted percentages, unweighted counts.*
Interpretation: *18.2% of individuals who reported being often discriminated against were unvaccinated. This group had 2.41 times higher odds of non-vaccination compared with those who reported never having been discriminated against*

Appendix 2: Factors associated with non-vaccination including the type and frequency of discrimination and frequency (logistic regression model)

Variables	%	OR	[95% CI]
Age			
18 - 24	8.5	1.33	[1.10 - 1.59]

25 - 34	10.8	2.50	[2.12 - 2.95]
35 - 44	10.1	2.23	[1.92 - 2.60]
45 - 54	8.4	1.58	[1.36 - 1.83]
55 - 64	5.6	1.29	[1.11 - 1.50]
65 - 74	3.3	ref	
75 +	3.1	0.70	[0.55 - 0.89]
Gender			
Men	7.7	1.16	[1.07 - 1.26]
Women	6.5	ref	
Standard of living			
D1	13.7	3.42	[2.83 - 4.14]
D2-D3	10.5	2.96	[2.47 - 3.54]
D4-D5	7.7	2.24	[1.88 - 2.68]
D6-D7	6.1	1.78	[1.50 - 2.12]
D8-D9	3.8	1.34	[1.13 - 1.58]
D10	2.4	ref	
Degree			
No diploma	7.2	1.03	[0.85 - 1.25]
Lower secondary	5.8	0.88	[0.74 - 1.04]
Technical diploma	8.4	0.99	[0.88 - 1.12]
High school diploma	8.7	ref	
Post secondary	7	0.79	[0.71 - 0.88]
Postgraduate degree	3.8	0.52	[0.44 - 0.60]
Racialized minority status			
Majority population	6.1	ref	
DROM population	15.6	2.21	[1.70 - 2.87]
Non-racialized immigrants	7.2	1.17	[0.99 - 1.39]

Racialized immigrants	16.1	1.85	[1.56 - 2.20]
Descendants of non-racialized immigrants	7.2	1.20	[0.96 - 1.52]
Descendants of racialized immigrants	9.8	1.19	[0.97 - 1.47]
Area size			
Rural	6.8	1.29	[1.16 - 1.43]
< 20 000 inh	7.4	1.21	[1.08 - 1.35]
20 000 - 100 000 inh	7.8	1.13	[1.00 - 1.29]
> 100 000 inh	6.9	ref	
Discrimination			
No discrimination	6	ref	
Other form(s) of discrimination than by a healthcare professional: *Sometimes*	10.2	1.51	[1.35 - 1.69]
Other form(s) of discrimination than by a healthcare professional: *Often*	15.8	2.14	[1.75 - 2.62]
Discrimination by a healthcare professional and other form(s) of discrimination: *Sometimes*	18.1	3.30	[2.58 - 4.21]
Discrimination by a healthcare professional and other form(s) of discrimination: *Often*	25.5	3.28	[2.28 - 4.71]
Discrimination by a healthcare professional only: *Sometimes*	16.8	4.07	[3.10 - 5.33]
Discrimination by a healthcare professional only: *Often*	27.2	3.49	[1.93 - 6.32]

Source: *EpiCoV Survey, Inserm-Drees, wave 4, Sept–Dec 2022. Weighted percentages, unweighted counts.*
Interpretation: *27.2% of individuals who were often discriminated against by a healthcare professional were unvaccinated. This group had 3.49 times higher odds of non-vaccination compared with those who had never been discriminated against.*

Appendix 3: Factors Associated with non-vaccination: Logistic regression with Interaction between discrimination and racialized minority status

Variables	%	OR	[95% CI]
Age			
18 - 24	8.2	1.28	[1.07 - 1.54]
25 - 34	11	2.48	[2.10 - 2.93]
35 - 44	9.8	2.20	[1.89 - 2.56]
45 - 54	8	1.54	[1.33 - 1.79]
55 - 64	5.7	1.27	[1.09 - 1.47]

65 - 74	3.3	ref	
75 +	3.2	0.71	[0.56 - 0.90]
Gender			
Men	7.4	1.15	[1.06 - 1.25]
Women	6.6	ref	
Standard of living			
D1	13.4	3.50	[2.90 - 4.23]
D2-D3	10.4	2.99	[2.50 - 3.58]
D4-D5	7.5	2.25	[1.88 - 2.68]
D6-D7	6%	1.78	[1.50 - 2.12]
D8-D9	3.8	1.34	[1.13 - 1.59]
D10	2.4	ref	
Degree			
No diploma	7.2	1.03	[0.85 - 1.25]
Lower secondary	5.7	0.88	[0.74 - 1.04]
Technical diploma	8	1.00	[0.88 - 1.12]
High school diploma	8.5	ref	
Post secondary	7.1	0.79	[0.71 - 0.89]
Postgraduate degree	3.8	0.51	[0.44 - 0.60]
Area size			
Rural	6.7	1.29	[1.17 - 1.43]
< 20 000 inh	7.4	1.21	[1.08 - 1.35]
20 000 - 100 000 inh	7.9	1.14	[1.01 - 1.29]
> 100 000 inh	6.6	ref	
Discrimination			
Majority population: never	5.2	ref	
Majority population: sometimes or often	11.8	2.01	[1.81 - 2.23]
DROM population: never	13.1	2.38	[1.72 - 3.31]
DROM population: sometimes or often	20.9	3.57	[2.34 - 5.44]
Descendants of non-racialized immigrants: never	6.2	1.09	[0.89 - 1.34]
Descendants of non-racialized immigrants: sometimes or never	13.1	2.81	[2.05 - 3.85]
Descendants of racialized immigrants: never	15.1	2.27	[1.83 - 2.82]
Descendants of racialized immigrants: sometimes or often	18	2.79	[2.16 - 3.61]
Non-racialized Immigrants: never	6.8	1.31	[1.01 - 1.70]
Non-racialized immigrants: sometimes or never	9.3	1.80	[1.10 - 2.94]
Racialized immigrants: never	9.5	1.37	[1.05 - 1.78]
Racialized immigrants: sometimes or often	10.3	1.89	[1.38 - 2.58]

Source: *EpiCoV Survey, Inserm-Drees, wave 4, Sept–Dec 2022. Weighted percentages, unweighted counts.*
Interpretation: *20.9% of individuals from the DROM population were unvaccinated. This group had 3.57 times higher odds of non-vaccination compared with those belonging to the majority population who had never been discriminated against.*

Appendix 4**:** Factors associated with non-vaccination among those reporting a reason for discrimination (logistic regression model)

Variables	%	OR	[95% CI]
Age			
18 - 24	8.1	1.41	[1.16 - 1.71]
25 - 34	10	2.67	[2.24 - 3.19]
35 - 44	9.5	2.38	[2.02 - 2.79]
45 - 54	7.6	1.61	[1.37 - 1.89]
55 - 64	5.2	1.31	[1.12 - 1.54]
65 - 74	3.1	ref	
75 +	2.9	0.73	[0.57 - 0.93]
Gender			
Men	7	1.11	[1.02 - 1.21]
Women	6.1	ref	
Standard of living			
D1	13.3	3.77	[3.08 - 4.62]
D2-D3	10	3.16	[2.61 - 3.82]
D4-D5	6.8	2.32	[1.92 - 2.81]
D6-D7	5.6	1.87	[1.56 - 2.25]
D8-D9	3.4	1.35	[1.13 - 1.62]
D10	2.1	ref	
Degree			
No diploma	7.2	1.10	[0.90 - 1.34]
Lower secondary	5.3	0.86	[0.72 - 1.03]
Technical diploma	7.6	1.00	[0.88 - 1.14]
High school diploma	8.1	ref	
Post secondary	6.3	0.77	[0.69 - 0.87]
Postgraduate degree	3.6	0.53	[0.45 - 0.62]
Racialized minority status			
Majority population	5.5	ref	
DROM population	15.1	2.26	[1.71 - 2.97]
Non-racialized immigrants	6.7	1.07	[0.88 - 1.30]
Racialized immigrants	15.3	1.96	[1.64 - 2.34]
Descendants of	6.7	1.21	[0.95 - 1.54]

non-racialized immigrants			
Descendants of racialized immigrants	10	1.35	[1.09 - 1.67]
Area size			
Rural	6.2	1.27	[1.14 - 1.42]
Rural	6.8	1.19	[1.05 - 1.34]
< 20 000 inh	7.1	1.13	[0.99 - 1.30]
20 000 - 100 000 inh	6.5	ref	
Type of discrimination			
No discrimination	6	ref	
By a healthcare professional only	11.1	1.62	[1.02 - 2.58]
By a healthcare professional and other form(s) of discrimination	16.4	2.25	[1.75 - 2.89]
Other form(s) of discrimination than by a healthcare professional	9.6	1.28	[1.13 - 1.46]

Source: *EpiCoV Survey, Inserm-Drees, wave 4, Sept–Dec 2022. Weighted percentages, unweighted counts.*
Interpretation: *11.1 % of individuals who reported being discriminated by a healthcare professional only were unvaccinated. This group had 1.62 times higher odds of non-vaccination compared with those who reported never having been discriminated against.*

Appendix 5: Factors associated with non-vaccination including trust in the Government's response to the epidemic (logistic regression model)

Variables	%	OR	[95% CI]
Age			
18 - 24	8.5	1.23	[1.02 - 1.49]
25 - 34	10.7	1.96	[1.65 - 2.32]
35 - 44	10.1	1.95	[1.67 - 2.28]
45 - 54	8.4	1.45	[1.24 - 1.69]
55 - 64	5.6	1.25	[1.07 - 1.46]
65 - 74	3.3	ref	
75 +	2.8	0.74	[0.58 - 0.95]
Gender			
Men	7.7	1.18	[1.09 - 1.29]
Women	6.5	ref	
Standard of living			
D1	13.8	2.70	[2.22 - 3.28]
D2-D3	10.2	2.24	[1.87 - 2.69]

D4-D5	7.7	1.82	[1.52 - 2.18]
D6-D7	6.1	1.51	[1.27 - 1.80]
D8-D9	3.8	1.20	[1.01 - 1.42]
D10	2.5	ref	
Degree			
No diploma	7.4	1.00	[0.82 - 1.21]
Lower secondary	5.6	0.87	[0.73 - 1.04]
Technical diploma	8.4	0.93	[0.82 - 1.06]
High school diploma	8.7	ref	
Post secondary	7	0.86	[0.77 - 0.96]
Postgraduate degree	3.8	0.62	[0.53 - 0.72]
Racialized minority status			
Majority population	6.1	ref	
DROM population	15.8	2.41	[1.83 - 3.15]
Non-racialized immigrants	7.2	1.20	[1.01 - 1.43]
Racialized immigrants	16.2	2.10	[1.76 - 2.51]
Descendants of non-racialized immigrants	7.2	1.50	[1.18 - 1.90]
Descendants of racialized immigrants	9.8	2.01	[1.61 - 2.49]
Area size			
Rural	6.8	1.24	[1.12 - 1.38]
< 20 000 inh	7.4	1.18	[1.05 - 1.33]
20 000 - 100 000 inh	7.8	1.13	[0.99 - 1.28]
> 100 000 inh	6.8	ref	
Trust in the Government's response to the epidemic			
Yes, completely	2.5	ref	
Yes, rather	3.3	1.80	[1.43 - 2.27]
No, rather not	9.1	5.81	[4.65 - 7.26]
No, not at all	17.4	12.9	[10.30 - 16.10]
Type of discrimination			
No discrimination	6	ref	
By a healthcare professional only	18.4	3.06	[2.37 - 3.96]
By a healthcare professional and other form(s) of discrimination	20.9	2.32	[1.87 - 2.87]
Other form(s) of discrimination than by a healthcare professional	11.1	1.32	[1.19 - 1.47]

Source: *EpiCoV Survey, Inserm-Drees, wave 4, Sept–Dec 2022. Weighted percentages, unweighted counts. Interpretation: 17.4% of individuals who reported not trusting the Government's response to the epidemic at all were unvaccinated. This group had 12.9 times higher odds of non-vaccination compared with those who reported complete trust in the Governm*
